# The role of PD-L1 in the radiation response and prognosis for esophageal squamous cell carcinoma related to IL-6 and T-cell immunosuppression

**DOI:** 10.18632/oncotarget.6861

**Published:** 2016-01-09

**Authors:** Miao-Fen Chen, Ping-Tsung Chen, Wen-Cheng Chen, Ming-Shian Lu, Paul-Yang Lin, Kuan-Der Lee

**Affiliations:** ^1^ Department of Radiation Oncology, Chang Gung Memorial Hospital at Chiayi, Chiayi, Taiwan; ^2^ College of Medicine, Chang Gung University, Chiayi, Taiwan; ^3^ Department of Hematology and Oncology, Chang Gung Memorial Hospital at Chiayi, Chiayi, Taiwan; ^4^ Department of Thoracic & Cardiovascular Surgery, Chang Gung Memorial Hospital at Chiayi, Chiayi, Taiwan; ^5^ Department of Pathology, Chang Gung Memorial Hospital at Chiayi, Chiayi, Taiwan

**Keywords:** PD-L1, esophageal SCC, IL-6, CD8+ T cell

## Abstract

The aim of this study was to assess the significance of programmed cell death 1 ligand 1 (PD-L1) in esophageal squamous cell carcinoma (ESCC) and its association with IL-6 and radiation response. Weretrospectively enrolled 162 patients with ESCC, and examined the correlation between PD-L1 levels and clinical outcomes in esophageal cancer patients. Furthermore, the human esophageal SCC cell line CE81T and TE2 were selected for cellular experiments to investigate the role of PD-L1 in T cell functions and radiation response. Here we demonstrated that PD-L1 expression was significantly higher in esophageal cancer specimens than in non-malignant epithelium. In clinical outcome analysis, this staining of PD-L1 was positively linked to the clinical T4 stage (*p*=0.004), development of LN metastasis (*p*=0.012) and higher loco-regional failure rate (*p*=0.0001). In addition, the frequency of PD-L1 immunoreactivity was significantly higher in IL-6-positive esophageal cancer specimens. When IL-6 signaling was inhibited *in vitro*, the level of PD-L1 is significantly down-regulated. PD-L1 is a significant predictor for poor treatment response and shorter survival. As demonstrated through *in vitro* experiments, Irradiation increased PD-L1 expression in human esophageal cancer cells. The inhibition of T cell functions including proliferation and cytotoxicity against tumor cells might be the mechanisms responsible to the role of PD-L1 in radiation response. In conclusion, PD-L1 is important in determining the radiation response and could predict the prognosis of patients with esophageal SCC. Therefore, we suggest inhibition of PD-L1 as a potential strategy for the treatment of esophageal SCC.

## INTRODUCTION

Esophageal cancer is one of the most common types of human cancer and a difficult gastrointestinal tumor to treat and cure [1]. Most patients who undergo curative treatment for esophageal cancer will eventually relapse and die as a result of their disease. Neoadjuvant chemoradiotherapy (CCRT) can increase the chance of R0 resection and have a survival benefit [2, 3]. Identification of the potential molecular markers for predicting the treatment response and understanding the molecular mechanisms underlying is important for the effective management and improving prognosis of esophageal cancer.

Tumor-induced immune suppression in cancer patients is a major issue that not only promotes tumor progression but also inhibits the efficiency of anti-cancer treatment [4, 5]. One of the major molecular regulators of tumor immune escape is programmed cell death 1 ligand 1 (PD-L1). PD-L1, a 40-kDa transmembrane protein belonging to the B7 family, negatively regulates T-cell signaling and inhibits T cell—mediated immune attack through binding to its receptor PD-1 on tumor-specific T cells [6, 7]. PD-L1 has been reported to be over-expressed in several human malignancies and link to poor prognosis and the resistance to anticancer therapies [8–10]. The issue to explore the key targets that can block PD-L1 expression and then enhance T-cell function in cancers has been brought into spotlight.

Increasing evidence suggests that the inflammatory response can be rerouted into a tumor-promoting direction association with poor prognosis in various type of cancer, and proinflammatory cytokines could promote PD-L1 expression in tumor microenvironment [10–12]. We previously observed that IL-6 was overexpressed in esophageal squamous cell carcinoma (ESCC), and their levels were positively correlated with disease progression [13]. At present, little is known about the potential regulatory function of IL-6 in the activation of PD-1/PD-L1 tumor immunopathology. The clinical efficacy was rather poor for ESCC might be due to the lack of knowledge of the tumor microenvironment including those concerning the PD-L1 in tumor and immune cells upon cytokine treatment. Therefore, we focused our work to assess the predictive value of PD-L1 expression in patients with ESCC. We also investigated the correlation between IL-6 and PD-L1 expression and their effects on tumor immune response to provide new insights into the development of immune-based therapy.

## RESULTS

### Expression of PD-L1 in ESCC

**Figure 1:**
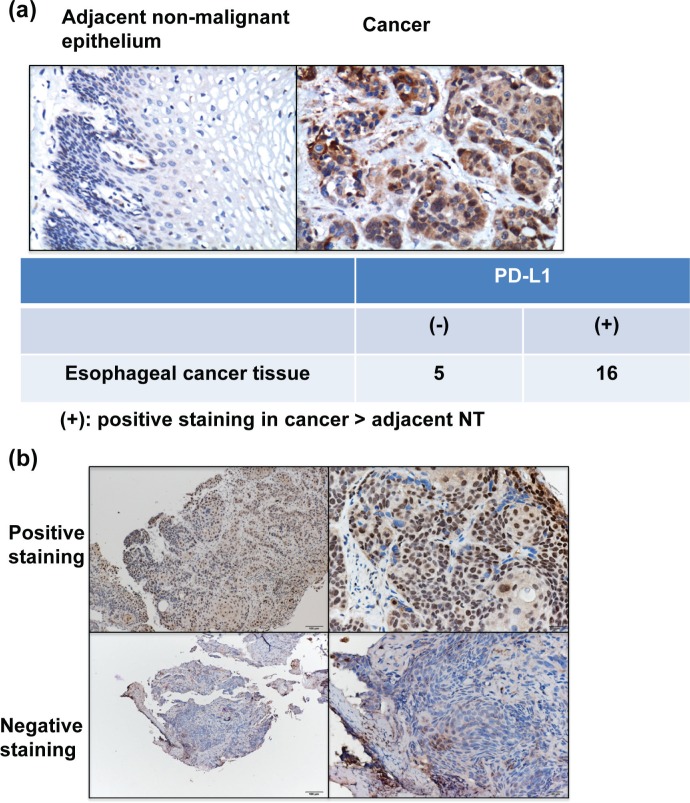
PD-L1 levels in esophageal SCC **a.** Representative images of IHC staining with an anti-PD-L1 antibody of esophageal cancer and adjacent non-malignant epithelium from TMA blocks. **b.** IHC staining with an anti-PD-L1 antibody of human esophageal cancer specimens. Images of representative slides are shown at magnifications of ×100 (left panel) and ×200 (right panel).

The IHC data for TMA slides demonstrated that PD-L1 was over-expressed at the membrane or in the cytoplasm (or both) of tumor cells in the surgically resected tumor specimens compared to adjacent non-malignant epithelial tissues (Figure 1a). Figure 1b showed the representative slides of positive staining and negative staining with anti-PD-L1 antibody for human esophageal cancer specimens at diagnosis. Of the 162 esophageal cancer tissues, 74 (45%) showed positive PD-L1 immunoreactivity (28% (25/88) in ≤T3 *versus* 50% (37/74) in T4, *P*=0.004). Furthermore, the positivity of PD-L1 was significantly associated with the risk of LN metastasis and developing loco-regional failure (Table 1).

**Table 1:** Clinico-pathological characteristics of esophageal cancer patients for immunohistochemical investigation

	No. of patients		*p* value
	IHC-PD-L1(−)	IHC-PD-L1(+)	
**patients**	88	74	
**Age**			
>=55.3y/0	43	39	0.588
<55.3y/o	45	35	
**Location**			
Upper third	18	18	0.174
Middle third	55	36	
Lower third	15	20	
**Tumor stage**			0.004*
<=T3	63	37	
T4	25	37	
**LN metastasis**			0.012*
negative	28	11	
positive	60	63	
**IL-6 staining**			<0.0001*
negative	60	19	
positive	28	55	
**Response to Neoadjuvant Tx**			<0.0001*
Response	75	40	
Non- response	13	34	
**Surgery s/p Neoadjuvant Tx**			0.228
Yes	29	18	
No	59	56	
**Local-regional Recurrence/persistent**			0.001*
No	40	16	
Yes	48	58	
**Distant metastasis**			0.208
negative	55	39	
positive	33	35	

Abbreviations: Neoadjuvant Tx = neoadjuvant chemoradiotherapy.

### Regulation of PD-L1 expression by IL-6 signaling in ESCC cells

**Figure 2:**
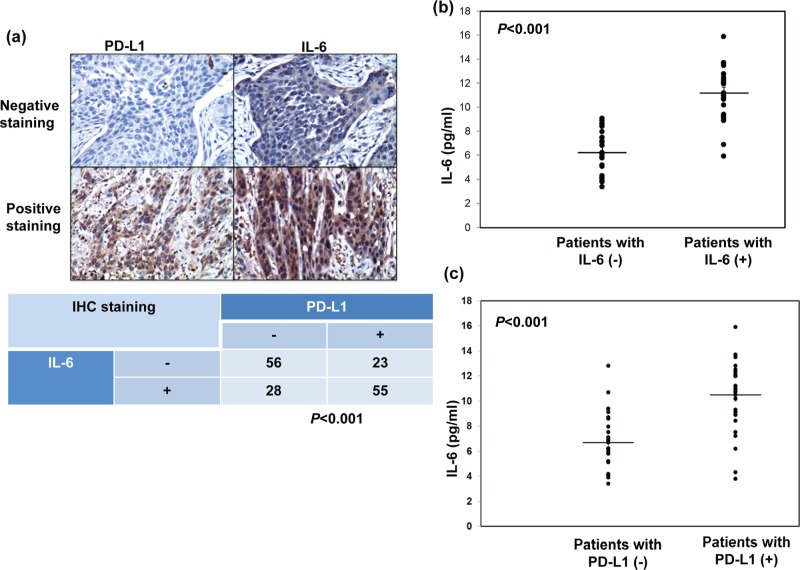
Correlation between PD-L1 and IL-6 levels **a.** IL-6 levels correlate positively withPD-L1 levels in human esophageal cancer specimens (*p*<0.001). Representative images of positive IL-6 and PD-L1 staining on slides from a selected tumor specimen, and representative negative staining for IL-6 andPD-L1 on slides from another tumor specimen, are shown. **b.** IL-6 levels measured using ELISA in plasma samples obtained from cancer patients with IL-6 negative staining (*n* = 27) and PD-L1 positive staing (*n* = 29) disease. The lines indicate the mean values (*p*<0.001). **c.** IL-6 levels measured using ELISA in plasma samples obtained from cancer patients with PD-L1 negative staining (*n* = 25) and PD-L1 positive staing (*n* = 31) disease. The lines indicate the mean values (*p < 0.001*).

**Figure 3:**
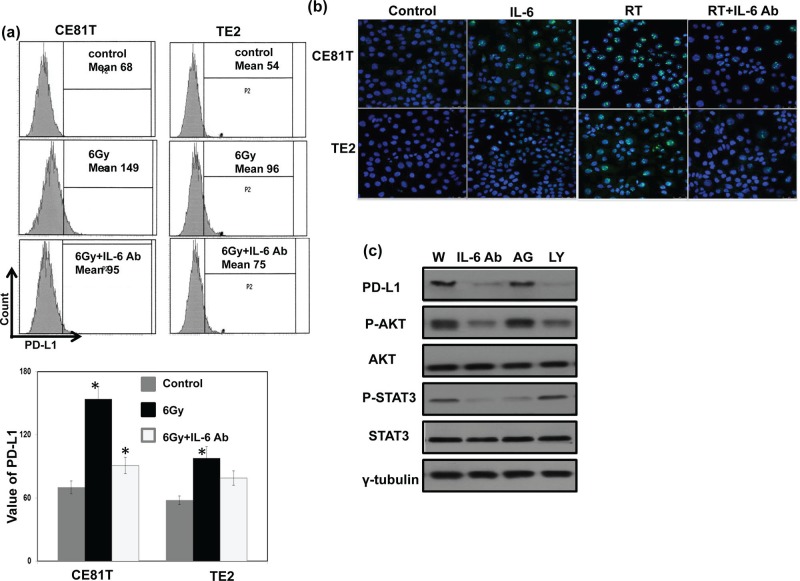
Role of IL-6 signaling on PD-L1 expression in human esophageal cancer The levels of PD-L1 were evaluated by **a.** FACS with PD-L1 antibody. Each column is shown as the means of 3 separate experiments; bars, SD.*, *P*<0.05; and **b.** IF staining for human esophageal cancer cells at 48h after IL-6 regulation *in vitro* (Blue, DAPI; Green, PD-L1). **c.** Effect of inhibited IL-6 signaling on PD-L1 protein levels as determined by immunoblotting (W, proteins were extracted from cells under control condition; IL-6Ab, proteins were extracted from cells incubated in the presence of 5 μg/ml IL-6 neutralizing antibodies for 48h; AG, proteins were extracted from cells incubated in the presence of 50 μM AG490 for 48 h; LY, proteins were extracted from cells incubated in the presence of 50 μM LY294002 for 48h).

We previously reported that IL-6 is a significant predictor for esophageal cancer. In the present study, we further investigate the correlation between IL-6 and PD-L1. As shown in Table 1 and Figure 2a, positive staining for IL-6 was evident in 51% of the 162 cancer specimens, and a significant positive correlation was found in cancer specimen that expressed PD-L1 and IL-6. Moreover, we examined the levels of IL-6 in the peripheral blood samples from the 56 SCC patients who diagnosed in 2013∼2014. The mean IL-6 level in esophageal SCC patients was 8.79±2.98 ng/ml. To further investigate the potential correlation of serum IL-6 levels with the levels of IL-6 and PD-L1 in esophageal cancer tissues, the 56 patients were divided into two groups according to their IL-6 or PD-L1 staining. There is positive link between the level of IL-6 in serum and tumor tissue. The mean IL-6 serum level in the IL-6 (−) group was 6.2±1.74 ng/ml, compared with 11.18±2.12 ng/ml in the IL-6 (+) group (Figure 2b). As shown in Figure 2c, the IL-6 levels were also significantly higher in patients with PD-L1 positive staining compared with PD-L1 (−) group (*p* < 0.001). Given the positive association between IL-6 and PD-L1 expression in ESCC tumors, we examined the expression of PD-L1 in esophageal cancer cell lines whose IL-6 was regulated. Flow cytometric analysis and IF data revealed that IL-6 neutralizing antibody significantly decreased the level of PD-L1 expression at the cell surface and the cytoplasm (Figure 3a–3b). Moreover, to investigate the pathway mediated the effect of IL-6 on PD-L1, we blocked STAT3 activation with JAK inhibitor and PI3K signaling using the specific inhibitor LY294002 in vitro. When PI3K pathway was inhibited, the decreases in PD-L1 protein levels were comparable to those induced by the IL-6-neutralizing antibody (Figure 3c). Therefore, it appears that activated IL-6-PI3K pathway might, at least in part, be responsible for the up-regulation of PD-L1 in esophageal cancer.

### Role of PD-L1 in the resistance of radiotherapy for esophageal cancer

**Figure 4:**
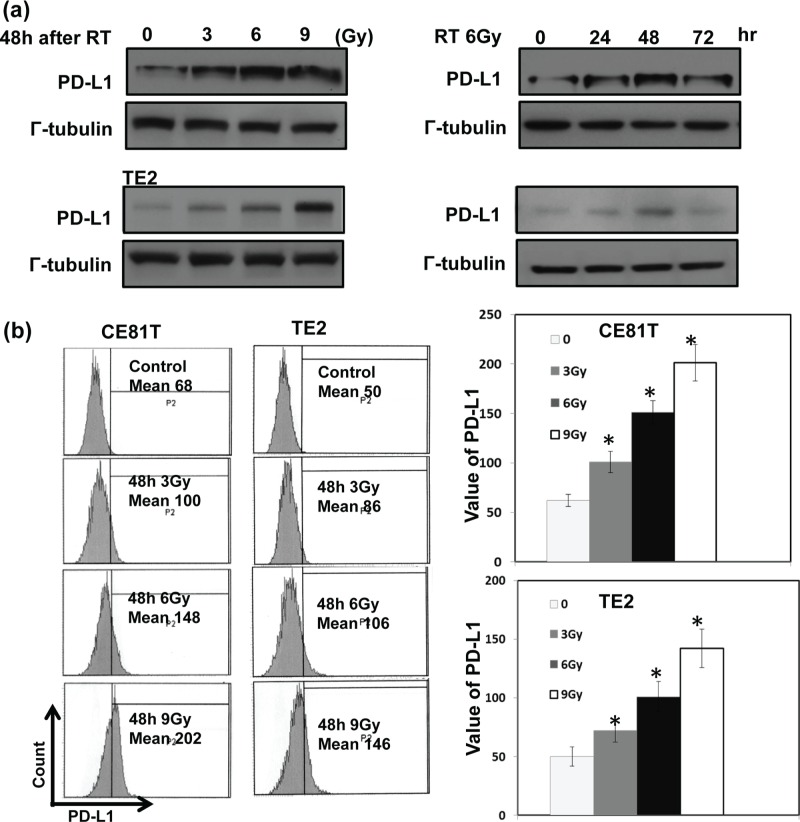

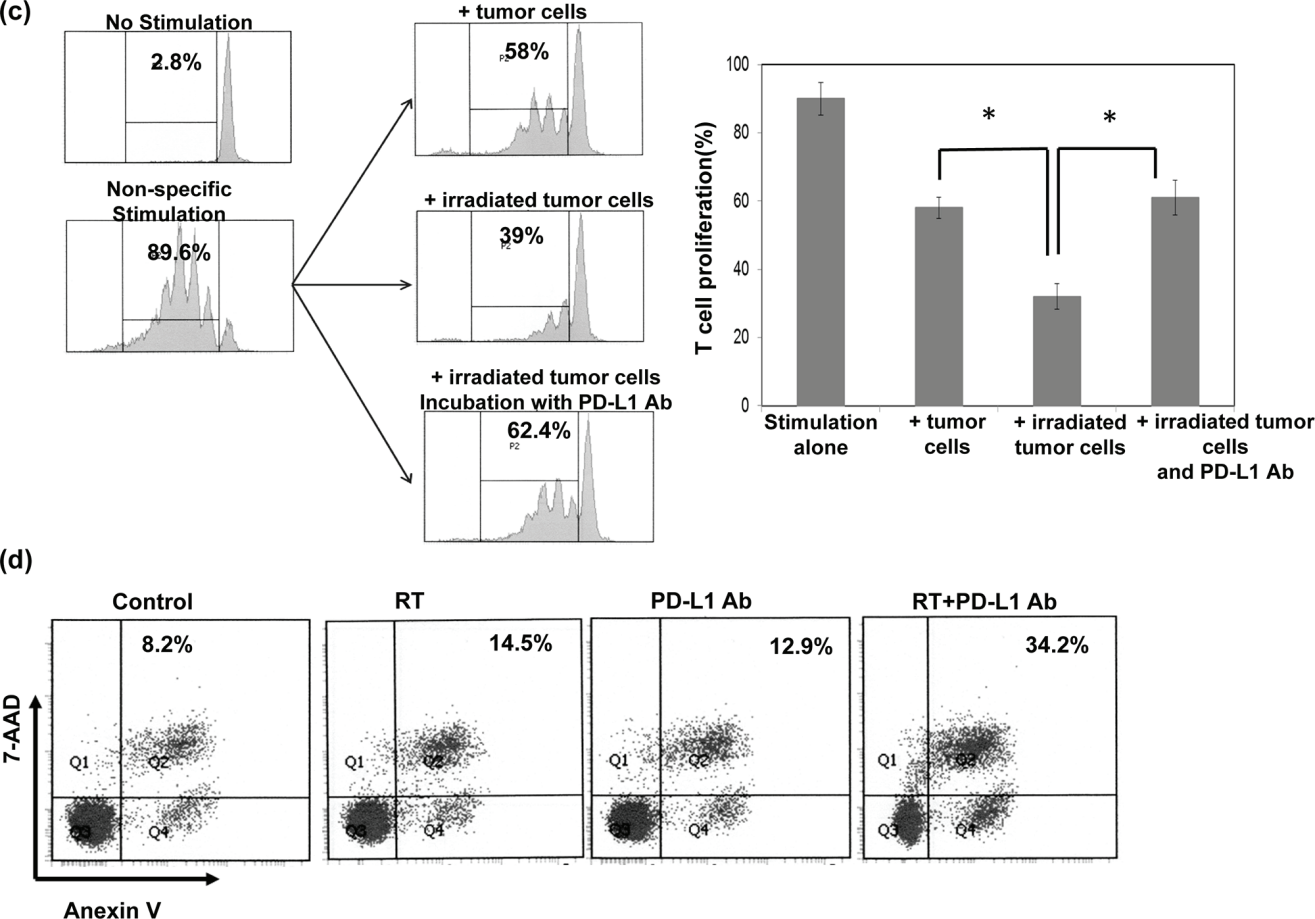
Correlation between irradiation, PD-L1 in cancer cells, and the function of cytotoxic T cells The levels of PD-L1 were evaluated by **a.** Western blot analysis and **b.** FACS with PD-L1 antibody for human esophageal cancer cells at indicated time after 6Gy irradiation or 48h after RT with 0, 3, 6, 9Gy *in vitro*. Representative slides are shown. Each column is shown as the means of 3 separate experiments; bars, SD.*, *P*<0.05. **c.** The effect of PD-L1 blockade on the suppressing ability of tumor cells for T cells proliferation was evaluated by FACS. Representative images and quantitative data are shown. Each column is shown as the means of 3 separate experiments; bars, SD.*, *P*<0.05; **d.** The effect of PD-L1 blockade on the CD8+ T cells cytotoxicity against cancer cells was evaluated by FACS. Representative images and quantitative data are shown.

For esophageal SCC, radiotherapy is a well-established therapeutic modality and provides survival benefits for responders. As shown in Table 1, the positive staining of PD-L1 significantly correlated with poor treatment response (35% (40/115) in responders *versus* 72% (34/47) in non-responders, *P*<0.001). Furthermore, 47 among these patients received esophagectomy after neoadjuvant CCRT, PD-L1 staininig linked with lower complete pathologic response rate (pCR) (16% (3/18) in PD-L1(+) patients versus 31% (9/29) in PD-L1 (−) patients)). The role of PD-L1 in radioresistance and its underlying mechanisms were further examined *in vitro*. As shown in Figure 4a–b, the level of PD-L1 in human esophageal cancer was increased by radiotherapy in the plasma membrane and cytoplasm of cancer cells when compared with nontreated cells. The increased level positively linked with the radiation dose. To directly test the functional consequences, the function of T cells against tumor cells was evaluated with or without blocking PD-L1. Irradiation increased the ability of tumor cells to suppress nonspecific stimuli (anti-CD3/CD28 antibody )-mediated T cell proliferation, and anti-PD-L1 attenuated the ability of irradiated tumor cells-mediated T cell suppression (Figure 4c). Inhibition of PD-L1 combined with irradiation resulted in increased tumor cytolysis compared with anti-PD-L1 monotherapy or irradiation alone when tumor cells co-cultured with sorting CD8+ cells from patients (Figure 4d).

### Correlation between the PD-L1 level and clinical outcome

**Figure 5:**
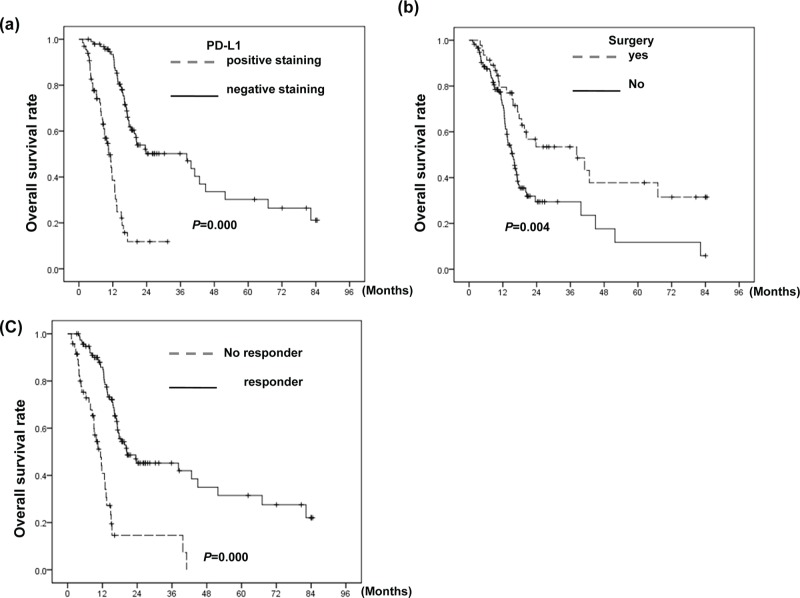
Correlation between PD-L1 level and clinical outcome Survival differences according to **a.** the positive staining of PD-L1; **b.** patients who underwent surgery or not; and **c.** the response to CCRT for all 162 patients. The PD-L1-positive group exhibited shorter survival than the PD-L1-negative group.

Table 2 and Figure 5 showed that PD-L1 was significantly correlated with a higher recurrence rate after curative treatment, and is a significant predictor for shorter survival. The median OS times were 39.7 and 11.4 months in patients whose tumor appearing PD-L1 negative staining and those with PD-L1 positive staining, respectively. In addition to PD-L1expression, poor treatment response, no tumor resection, and advanced T- stage were significantly associated with poor OS and DFS. The positive PD-L1 staining still had the predictive value for OS by multivariate analysis.

**Table 2A:** Univariate analysis to determine factors associated with prognosis

Variables	*P* value for Overall survival	*P* value for Disease-free survival
Clinical T stage	0.013*	0.001*
Tumor resection	0.004*	0.000*
Positive staining for PD-L1	0.000*	0.000*
Clinical N stage	0.366	0.086
Local-regional Recurrence	0.000*	0.000*
Distant metastasis	0.034*	0.002*
Treatment response	0.000*	0.000*

**Table 2B:** Multivariate analysis to determine molecular markers associated with prognosis (OS) of patients

Variables	Odd ratios	95% confidence interval	*p*
PD-L1 staining	0.380	0.200-0.648	0.001*
T stage	0.937	0.583-1.506	0.787
Tumor resection	1.796	1.080-2.989	0.024*
LN involvement	1.106	0.647-1.891	0.713
Treatment response	0.403	0.230-0.707	0.002*
Recurrence and/or distant metastasis	0.387	0.192-0.703	0.003*

## DISCUSSION

Malignant tumors possess mechanisms for evading host immune responses. A novel mechanism that tumor may evade host immune response through the expression of PD-L1. PD-L1 expression has been found in various human cancers, and was associated with patients' prognoses [17–19]. In the present study, we investigate the clinical significance of PD-L1 and its link with IL-6 signaling in ESCC. The IHC data revealed that PD-L1 expression on esophageal cancer tissue was significantly higher than those noted in adjacent non-malignant tissues. The frequency of PD-L1 positive staining was significantly increased with advanced T4 stage and positive lymph node metastasis. Furthermore, by univariate analysis, enhanced expression of PD-L1, advanced stage, without receiving surgery and poor response to CCRT were significantly associated with a shorter survival, and PD-L1 still possessed predictive power for overall survival in multivariate analysis. Based on the data, PD-L1 may be a critical factor to promote tumor growth and invasion in esophageal cancer.

PD-L1/PD-1 has been shown to be an important mechanism underlying T-cell dysfunction during chronic inflammation and chronic infection [20–22]. Several cytokines and the activation of PI3K and STAT3 pathway have been reported to increase PD-L1 expression in some cancers [10, 23–25]. IL-6 is the main cytokine influencing the inflammatory response in humans and a major activator of PI3K and STAT3 signaling pathways [26, 27]. IL-6 signaling has been implicated in regulation of tumor growth and metastatic spread, and its level could be correlated with poor prognosis in different cancers [26, 28, 29]. Serum IL-6 levels have been shown to be higher in patients with esophageal carcinoma than in healthy controls [30–32]. IL-6 levels in tumor tissues is also higher than adjacent normal esophageal tissues [31–33]. The correlation between IL-6 levels and tumor progression indicates that IL-6 in tumor tissues plays a pivotal role in the pathological behavior of esophageal carcinoma. We have showed that IL-6 could be a significant predictor for clinical stage and prognosis of esophageal SCC [13]. Therefore, the present study was undertaken to discover the distribution and regulation of PD-L1 in esophageal SCC by IL-6 in the tumor microenvironment. By IHC and ELISA analyses using clinical specimen, there was a positive correlation between the expression levels of PD-L1 and IL-6. We also showed that expression of PD-L1 was obviously decreased when blocking IL-6 with neutralizing antibody in vitro. Furthermore, our data revealed that the inhibition of the PI3K pathway could attenuated the expression of PD-L1 comparable to those induced by the IL-6-neutralizing antibody. Therefore, it is likely that PI3K activation plays a role in transmitting IL-6 signals to downstream targets that regulate the PD-L1 expression in ESCC. Accordingly, we suggested IL-6 is critical in the up-regulation of PD-L1 expression in esophageal cancer.

For ESCC, treatment response is an independent prognostic factor. The identification and inhibition of key drivers of immunosuppression have the potential to improve patient outcome combined with radiotherapy. PD-L1 expression has been reported to link with the resistance to anticancer therapies [8, 9]. Our clinical data revealed enhanced expression of PD-L1 was significantly associated with a lower response to CCRT and a greater risk of loco-regional failure. In vitro data of upregulation of PD-L1 observed in the irradiated esophageal cancer cells suggested that alteration of PD-L1 levels in the tumor microenvironments may play a role in the radiation response of esophageal cancer.

It is known that infiltration of T cells into tumors correlates with improved prognosis in several types of GI cancers [34–36]. Furthermore, the presence of negative regulatory factors, such as regulatory T cells and myeloid-derived suppressor cells, which can inhibit antitumor T-cell responses, correlates with a poor prognosis cancers [34, 37]. For esophageal cancer, the presence and the number of CD 8+ tumor-infiltrating T lymphocytes has been interpreted as having a positive impact on overall survival [38–40]. PD-L1 is thought to limit T-cell function within tissue sites by binding its receptor, PD-1, found on activated T cells [41, 42]. Upregulation of the PD-1/PD-L1 axis was observed to suppress cytotoxic action of T cell, which may be the cause of tumor evading host immune responses and incomplete tumor cell killing after irradiation [41–43]. Therefore, we investigated the role of PD-L1 of esophageal cancer cells in the functionality of cytotoxic T cells in vitro. The data revealed that tumor cells' PD-L1 expression was triggered by radiation. Blockade of PD-L1 effectively inhibit CD8+ T cells cytotoxicity against irradiated esophageal cancer cells. We demonstrated that the increase in PD-L1 expression on irradiated esophageal cancer cells was associated with a decreased T cell proliferation upon co-culture experiments, and anti-PD-L1 had reduced the suppressive ability for T cell proliferation. Base on the data, we suggest the inhibition of T-cell activation by increased PD-L1 responsible to the radioresistance of esophageal cancer, at least a part.

Our study has some limitations. First, we examined PD-L1 expression mainly in biopsy tissue specimen to evaluate its prognostic value. Biopsies only sampled a small esophageal volume, which might result in undersampling of esophageal cancer tissue. Also, to examine the prognostic value of PD-L1 in esophageal cancer patients, it is a retrospective analysis of a population with different stages from a single institution. Therefore, the issue should be best answered in context of a prospective study in a more patient population.

These findings indicate that PD-L1-positive esophageal cancer provides a suitable microenvironment for the development of tumor growth and treatment resistance mediated by induction of activated IL-6 signaling. Novel immune-based therapies for the treatment of cancer are currently under development. Therefore, targeting PD-L1 could be a promising strategy for the treatment of esophageal cancer.

## MATERIALS AND METHODS

### Patient characteristics

This study was approved by the institutional review board of our hospital. Patients who did not comply with the treatment regimen and those who received surgery alone for early-stage esophageal cancer were excluded from the study. A total of 162 patients with ESCC were enrolled in the study. The curative treatment for esophageal cancer included neoadjuvant CCRT combined with surgery or definitive CCRT according to the guidelines proposed by oncology team at our hospital. On completion of neoadjuvant CCRT, patients underwent a repeat CT scan and endoscopic examination to determine the response to treatment. Specimens collected from the 162 patients at diagnosis were subjected to immunochemical analysis. The main end points were overall survival (OS), disease-free survival (DFS) and treatment response. Survival probability was analyzed statistically using the Kaplan—Meier method. Significant differences between groups were assessed using the Spearman-rank test. Multivariate analyses were performed using a Cox regression model for survival.

### Immunohistochemical staining (IHC)

Formalin-fixed, paraffin-embedded tissues from 162 patients with esophageal cancer were subjected to IHC staining. Dissected esophageal cancer specimens from 21 of these patients were converted into tissue microarray (TMA) blocks using an AutoTiss 1000 arrayer (Ever BioTechnology, Canada). The TMA block contained esophageal SCCs and the adjacent non-malignant epithelium. The quality of the TMA slides was confirmed by the pathologist using hematoxylin- and eosin-stained slides. The IHC data for the specimens were assessed using the semi-quantitative immunoreactive score (IRS), as described previously [13]. For the evaluation, all tumor cells in the slides were taken into consideration. An ROC curve was calculated and best cut-off points were determined in esophageal cancer cells compared with adjacent non-malignant tissue. An IRS scoring grade of >= 2 was considered positive IHC scoring

### Enzyme-linked immunosorbent assay analysis of IL-6 levels

Peripheral blood (PB) samples were obtained from 56 of these patients before treatment. IL-6 levels in human serum samples were analyzed using an IL-6 human Quantikine ELISA kit (R&D system). The samples were frozen and stored before assaying.

### Cell culture and reagents

The human cancer cell lines CE81T, which is derived from a well-differentiated esophageal SCC and obtained from Bioresource Collection and Research Center [14], and TE2, which is derived from a poorly-differentiated esophageal SCC and provided by Dr S-H Li [15], were used in the present study. The cytokine- IL-6, IL-6 neutralizing antibody and PD-L1-neutralizing antibody were purchased from R&D Systems (Minneapolis, MN, USA) and Biolegend (San Diego, CA), respectively.

### Flow cytometric analysis

To obtain single-cell suspensions, tumor tissues were digested by 1 mg/ml collagenase IV (Sigma-Aldrich) and 0.2 mg/ml DNase I (Sigma-Aldrich) for 45 minutes at 37°C. Cells were blocked and then stained with antibodies against PD-L1. For apoptosis assays, tumor cells were stained with antibodies against 7-AAD and annexin V after the removal of CD8+ cells. To isolate cytotoxic T cells from peripheral blood mononuclear cells (PBMC) in the patients, multicolor fluorescence-activated cell sorting (FACS) was performed using a FACS caliber flow cytometer (BD Biosciences). The human cytotoxic T cells subset characterized as CD3^+^CD8^+^ was sorted from freshly obtained PB.

### T-cell suppression assay

The suppressive function of tumor cells was measured by their ability to inhibit the proliferation of autologous T cells in the following Suppression Assay, as described previously [16]. The isolated CD8+T cells from patients were CFSE-labeled (3 μM, Sigma) and seeded in 96-well plates (at 2 × 105 cells/well ) with tumor cells with 1:1 ratio. T cell proliferation was induced by anti-CD3/CD28 stimulation beads (Invitrogen, Carlsbad, CA). Suppression Assay was analyzed by flow cytometry for T cell proliferation after three days.

### Statistical analysis

The significance of differences between samples was determined using Student's t-tests. Data are presented as the means ± standard error of the mean (SEM). All experiments, comprising three replicates, were performed at least twice independently. A probability level of p < 0.05 was adopted throughout to determine statistical significance, unless otherwise stated.
